# miR-34b-3p-mediated regulation of STC2 and FN1 enhances chemosensitivity and inhibits proliferation in cervical cancer: miR-34b-3p targets *STC2* and *FN1* to enhance chemosensitivity

**DOI:** 10.3724/abbs.2024009

**Published:** 2024-03-13

**Authors:** Shanshan Jin, Wenting Wang, Xinrui Xu, Zhaowei Yu, Zihan Feng, Jun Xie, Huimin Lv

**Affiliations:** 1 Department of Biochemistry and Molecular Biology Shanxi Key Laboratory of Birth Defect and Cell Regeneration Key Laboratory for Cellular Physiology of Ministry of Education Shanxi Medical University Taiyuan 030001 China; 2 Shanxi Bethune Hospital Shanxi Academy of Medical Sciences Tongji Shanxi Hospital Third Hospital of Shanxi Medical University Taiyuan 030032 China; 3 Tongji Hospital Tongji Medical College Huazhong University of Science and Technology Wuhan 430030 China

**Keywords:** miR-34b-3p, chemoresistance, cervical cancer, stanniocalcin 2 (STC2), fibronectin 1 (FN1), cisplatin

## Abstract

Dysregulation of microRNA (miRNA) expression in cancer is a significant factor contributing to the progression of chemoresistance. The objective of this study is to explore the underlying mechanisms by which miR-34b-3p regulates chemoresistance in cervical cancer (CC). Previous findings have demonstrated low expression levels of miR-34b-3p in both CC chemoresistant cells and tissues. In this study, we initially characterize the behavior of SiHa/DDP cells which are CC cells resistant to the chemotherapeutic drug cisplatin (DDP). Subsequently, miR-34b-3p mimics are transfected into SiHa/DDP cells. It is observed that overexpression of miR-34b-3p substantially inhibits the proliferation, migration, and invasion abilities of SiHa/DDP cells and also enhances their sensitivity to DDP-induced cell death. Quantitative RT-PCR and western blot analysis further reveal elevated expression levels of STC2 and FN1 in SiHa/DDP cells, contrary to the expression pattern of miR-34b-3p. Moreover, STC2 and FN1 contribute to DDP resistance, proliferation, migration, invasion, and decreased apoptosis in CC cells. Through dual-luciferase assay analysis, we confirm that STC2 and FN1 are direct targets of miR-34b-3p in CC. Finally, rescue experiments demonstrate that overexpression of either STC2 or FN1 can partially reverse the inhibitory effects of miR-34b-3p overexpression on chemoresistance, proliferation, migration and invasion in CC cells. In conclusion, our findings support the role of miR-34b-3p as a tumor suppressor in CC. This study indicates that targeting the miR-34b-3p/STC2 or FN1 axis has potential therapeutic implications for overcoming chemoresistance in CC patients.

## Introduction

Cervical cancer (CC) is a major global health concern and is the fourth most common malignancy in females [1]. Despite advancements in vaccination, screening, and treatment, the 3- to 5-year surviving ratio in developing countries remains less than 50% [2]. Platinum-based combination chemotherapy is an important adjuvant treatment for CC [3]. However, the development of chemoresistance to the first-line chemotherapeutic drug cisplatin (DDP) has significantly impacted patient prognosis [4]. The mechanisms underlying DDP-resistance in CC are still not fully understood, highlighting the need for a better understanding of the mechanism in CC DDP-resistance to develop more effective therapies.

MicroRNAs (miRNAs) are small noncoding RNA molecules that function as negative regulators of mRNA expression [5]. They exert post-transcriptional regulation through complementary base pairing with target gene mRNAs [6]. One miRNA can target multiple genes, and multiple miRNAs also can regulate the same target gene [7]. While previous studies have investigated the function of miR-34b-3p in the development of various cancers, such as renal cell carcinoma and endometrial cancer [8], its specific involvement in CC chemoresistance remains poorly understood.

In our previous study, we found that the expression of STC2 is inversely correlated with that of miR-34b-3p in SiHa/DDP cells and that miR-34b-3p can target *STC2*, suggesting that miR-34b-3p may mediate CC resistance through STC2 [9].

In the present study, we identified fibronectin 1 ( *FN1*) as another target gene of miR-34b-3p. To further investigate the functions and potential mechanism of miR-34b-3p in CC chemoresistance, we examined how miR-34b-3p modulates CC chemoresistance by targeting both *STC2* and *FN1*. Our findings provide novel insights into CC chemoresistance and new clues for discovering screening markers and developing therapeutic strategies for CC treatment.

## Materials and Methods

### Establishment of DDP-resistant SiHa cells

The SiHa cell line, derived from human cervical squamous cell carcinoma, was obtained from National Collection of Authenticated Cell Cultures (Shanghai, China). The cells were cultured in DMEM (Gibco, Carlsbad, USA) supplemented with 10% fetal bovine serum (FBS; BI, Kibbutz Beit Haemek, Israel), 100 U/mL penicillin, and 0.1 mg/mL streptomycin (Solarbio, Beijing, China) at 37°C in cell incubator(Eppendorf, Hamburg, Germany) with 5% CO _2_.

To establish the DDP-resistant cell line (SiHa/DDP), the parental SiHa cell line was subjected to increasing concentrations of DDP in a stepwise manner [9]. The SiHa cells in the logarithmic growth phase were seeded into a 6-cm Petri dish. When the cell density reached 40%‒50%, the medium was replaced by DMEM supplemented with 0.5 μM DDP, 10% FBS, 100 U/mL penicillin, and 0.1 mg/mL streptomycin. The cells were then cultured at 37°C in cell incubator with 5% CO _2_ for 48 h. Once cell growth was inhibited, the medium was replaced by DDP-free medium until the cells resumed normal proliferation. Subsequently, medium containing 0.5 μM DDP was added again, and the cells were cultured for 48 h. This process was repeated until the cells stably proliferated in the presence of DDP. The concentration of DDP was increased incrementally (0.5, 1, 2, 4, 6, and 8 μM). The induction process was considered complete when the half-maximal inhibitory concentration (IC _50_) of SiHa/DDP cells was 5-fold greater than that of SiHa cells under 8 μM DDP treatment, and the cells exhibited stable DDP resistance and proliferation. To maintain drug resistance, DDP was supplemented at a concentration of 8 μM in the culture medium of SiHa/DDP cells.

### Cell transfection

All plasmids and small interfering RNAs (siRNAs) used for transfection were provided by GenePharma (Shanghai, China). The sequences of siRNAs are shown as follows: siSTC2: 5′-CAGAATACAGCGGAGATCCAGCACT-3′, siFN1: 5′-TTGTTATGACAATGGAAAACACT-3′, and miR-34b-3p mimics: 5′-CAATCACTAACTCCACTGCCAT-3′. The SiHa/DDP cells in the logarithmic growth phase were inoculated at a concentration of 1×10 ^6^ cells per well in 6-well plates and cultured for 24 h prior to transfection. Transfection was performed using Lipofectamine 8000 (Beyotime, Shanghai, China) when the cells reached 70%‒80% confluence. The pcDNA recombinant vector or siRNA was diluted in DMEM at 2.5 μg per well and mixed with RNA/DNA-Lipofectamine 8000. The transfection solution was added dropwise to the cells. Transfection efficiency was assessed by qRT-PCR and western blot analysis after 48 h and 72 h, respectively.

### Cell Counting Kit-8 (CCK-8) assay

Cell viability was determined by CCK-8 assay. SiHa/DDP cells were seeded in 96-well plates at 1×10 ^4^ cells per well in complete medium and grown for 24 h. Afterward, fresh medium was introduced to replace the existing medium containing different concentrations of DDP (0, 16, 32, 64 , 128, 256, or 512 μM). After incubation for 24 h, 10 μL of CCK-8 reagent (Solarbio, Beijing, China) was added to each well, and the plates were incubated for an additional 2 h. The absorbance of each well was measured at 450 nm to assess cell viability. The experiment was repeated at least three times and the IC _50_ values were calculated.

### Colony formation assay

Cells were seeded into a 6-well plate at a density of l×l0 ^3^ cells per well and incubated in an incubator at 37°C with 5% CO _2_ for 10‒14 days until visible colonies formed. To maintain cell viability, the medium was substituted every 2‒3 days. Prior to fixation, the cells were washed with PBS and then fixed in 4% polyformaldehyde for 30 min. Staining was performed using crystal violet. Colony formation rates were calculated by counting the number of colonies of at least 50 cells. Each experiment was conducted at least three times.

### Wound healing assay

The cells were seeded in a 6-well plate at a density of 5×10 ^5^ cells per well and incubated at 37°C for 6 h. A 100-μL pipette tip was used to create a wound in the middle of each well, after which the cells were washed and further incubated in serum-free culture medium. After 24 and 48 h, the migration distance of cell scratch area was observed under a microscope and photographed. Images of the wounds were captured, and the wound area was determined using ImageJ software.

### Transwell assay

First, Matrigel (356234; Corning, New York, USA) and serum-free medium was mixed at 1:8, and the upper compartment of the transwell was filled with 60 μL of diluted Matrigel. The transwell was then incubated at 37°C with 5% CO _2_ for 3 h to allow the Matrigel to polymerize into films and coat the polycarbonate microporous membrane. Next, 1×10 ^5^ cells were seeded into the upper compartment, which was filled with serum-free medium, while the lower compartment was filled with medium containing 10% FBS. After incubation for 24 h, the number of invaded cells was determined after fixation, staining, and counting the cells in three random fields per filter.

### Apoptosis assay

The cells were harvested and resuspended in 1× binding buffer to obtain a cell suspension of 5×10 ^6^ cells/mL. The cells were then mixed with 5 μL of Annexin V and 5 μL of 7-AAD (KGA108; KeyGEN, Shanghai, China) and incubated at room temperature in the dark for 15 min. Apoptosis detection was carried out with flow cytometer (BD FACSCanto II; BD Biosciences, Franklin Lakes, USA) following standard procedures.

### qRT-PCR

Total RNA was extracted from CC cells and subjected to reverse transcription for miRNA and mRNA using the Mir-X miRNA First-Strand Synthesis kit (TaKaRa, Dalian, China) and the PrimeScript™ RT Reagent kit with gDNA Eraser (TaKaRa), respectively, following the manufacturer’s instructions. Subsequently, qRT-PCR was performed using the TB Green® Premix Ex Taq™ II kit (TaKaRa) and the QuantStudio™ 3 Real-Time PCR System (Thermo Fisher, Waltham, USA). The expression levels of miRNAs and mRNAs were normalized to those of *U6* and *β-actin*, respectively. Each experiment was repeated three times, and the relative expression was calculated using the 2 ^–ΔΔCt^ method. Table 1 lists the sequences of primers used, which were obtained from Sangon (Shanghai, China).

**Table:** **Table 1** Sequences of primers used for qRT-PCR

Gene	Primer sequence (5′→3′)
*hsa-miR-34b-3p*	Forward: AGGCAGTGTAGTTAGCTGATTGC Reverse: TGGTGTCGTGGAGTCG
*STC2*	Forward: GGGTGTGGCGTGTTTGAATG Reverse: CTTGAGGTAGCATTCCCGCT
*U6*	Forward: GCTTCGGCAGCACATATACTAAAAT Reverse: CGCTTCACGAATTTGCGTGTCAT
*GAPDH*	Forward: ACCACAGTCCATGCCATCAC Reverse: TCCACCACCCTGTTGCTGTA
*FN1*	Forward: TGGGCAACTCTGTCAACGAA Reverse: GAGCAAATGGCACCGAGATA
*β-Actin*	Forward: GGGACCTGACTGACTACCTC Reverse: TCATACTCCTGCTTGCTGAT
*ERCC*	Forward: TACAAGGCCTATGAGCAGAAACCAG Reverse: ACTTCACGGTGGTCAGACATTCAG
*P-gp*	Forward: TTGATTGACAGCTACAGCACGGAAG Reverse: TTCTTCACCTCCAGGCTCAGTCC
*MDR*	Forward: AGGCCAACATACATGCCTTCATC Reverse: GCTGACGTGGCTTCATCCAA

### Western blot analysis

CC cells were harvested and lysed in RIPA lysis buffer (Solarbio) and the protein concentration was determined using a BCA Protein Assay kit (Solarbio). The lysate was then separated by 10% SDS-PAGE and transferred to a polyvinylidene fluoride (PVDF) membrane. Subsequently, the PVDF membranes were blocked with 5% nonfat milk for 1 h, followed by incubation overnight at 4°C with primary antibodies against STC2 (1:1000; Abcam, Cambridge, UK) and FN1 (1:1000; Abcam). Then membranes were incubated with HRP-conjugated secondary antibody (1:5000; Abmart, Shanghai, China) for 2 h at room temperature. Finally, protein bands were visualized using enhanced chemiluminescence (ECL) reagents (Abbkine, Wuhan, China).

### Dual-luciferase reporter assay

To investigate the relationship between miR-34b-3p and *STC2* or *FN1*, a dual-luciferase reporter assay was employed. CC cells were cotransfected with miR-34b-3p and either pmirGLO-STC2 (wild-type, WT) or pmirGLO-STC2 (mutant type, Mut) using Lipo 3000 (Invitrogen, Carlsbad, USA). After 48 h, the luciferase activities were measured using a Dual-Luciferase Reporter Gene Assay System (E1910; Promega, Madison, USA) according to the manufacturer’s protocol.

### RNA-sequencing and bioinformatics analysis

miRNA sequencing and bioinformatics analysis were performed basically according to the workflow as previously described [9]. Briefly, total RNA was isolated from SiHa and SiHa/DDP cell lines using Trizol reagent. Then, a small-RNA sequencing library was generated from the qualified total RNA sample using the NEBNext Multiplex Small RNA Library Prep Set for Illumina. It was sequenced on an Illumina NextSeq 500 platform after a quality control procedure. The clean miRNA reads obtained with Trimmomatic (v0.36) were aligned to human reference genome (hg19). Known and unknown miRNAs were identified by mirBase (v21) and mirDeep2 (v2.0.0.8), respectively. Finally, differential miRNA expressions were analyzed using an R package edgeR (v3.18.1). A threshold to filter out the exponential component was set as the p-value less than 0.05 and fold change larger than 2 or less than 0.5. Additionally, miRanda (v3.3a) was used to predict miRNA-mRNA interactions in animals.

### Statistical analysis

All the experiments were performed three times independently. Data were analyzed utilizing SPSS 23.0 software or GraphPad Prism 8.0. Differences between two groups were evaluated by Student’s *t*-test. *P*<0.05 was considered statistically significant.

## Results

### Characterization of DDP resistance and enhanced malignant properties in SiHa/DDP cells

**Figure 1:**
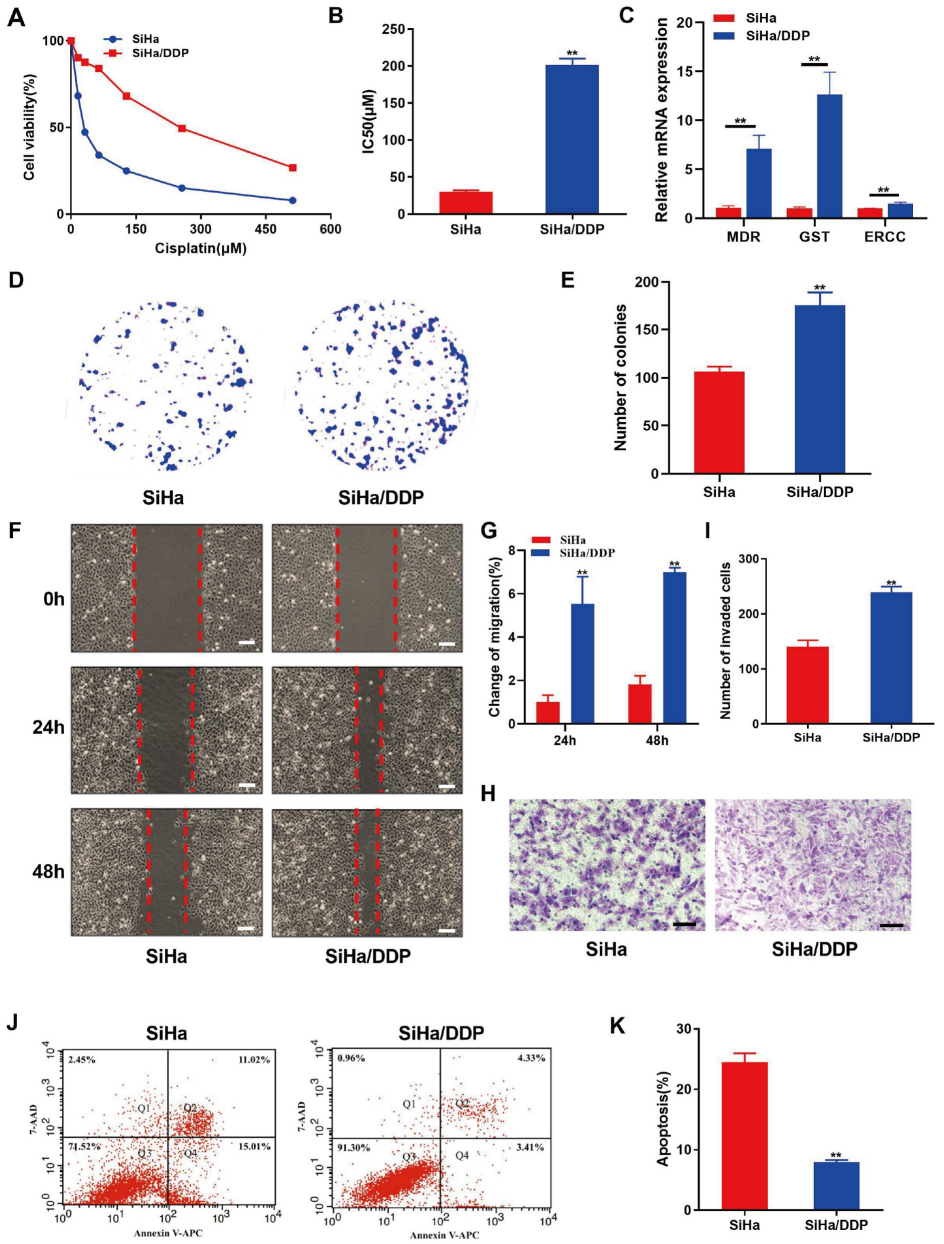
Figure 1 Comparison of the biological functions of SiHa cells and SiHa/DDP cells (A,B) The viability (A) and IC50 (B) of SiHa and SiHa/DDP cells were determined via CCK8 assay. (C) The mRNA expressions of MDR, GST and ERCC in SiHa and SiHa/DDP cells were measured by qPCR analysis. (D,E) The colony formation ability of SiHa cells and SiHa/DDP cells was determined by colony formation assay. (F–I) The migration (F,G) and invasion (H,I) abilities of SiHa cells and SiHa/DDP cells were detected by wound healing and transwell assays, respectively. (J,K) Cell apoptosis of SiHa and SiHa/DDP cells was evaluated via flow cytometry. Colony formation, migration, invasion and apoptosis experiments were all performed after treatment with 10 μM DDP. Scale bar: 50 μm. **P<0.01 by Student’s t test.

To determine the DDP resistance of SiHa/ DDP cells, CCK8 assay was used to determine the inhibitory effect of DDP at various concentrations on both the parental SiHa and SiHa/DDP cells. The results revealed significantly greater drug resistance in the SiHa/DDP cells than in the SiHa cells, with a more than 7-fold increase ( Figure 1A,B). Subsequently, the expression levels of *MDR*, *GST*, and *ERCC* genes were investigated, which are associated with resistance, in both cell types. Interestingly, the expressions of these three genes was significantly upregulated in the SiHa/DDP cells ( Figure 1C). Additionally, the behaviors of these two CC cell lines were examined. Our results showed that the SiHa/DDP cells exhibited significantly greater colony formation, migration, and invasion abilities than the SiHa cells under the the same concentration of DDP ( Figure 1D‒I). Remarkably, upon treatment with the same concentration of DDP, the percentage of apoptotic cells in the SiHa/DDP cells was significantly reduced ( Figure 1J‒K). Collectively, these findings indicate the successful establishment of a DDP-resistant CC cell line with malignant biological characteristics.

### Role of miR-34b-3p in reducing DDP resistance and malignant properties in CC

**Figure 2:**
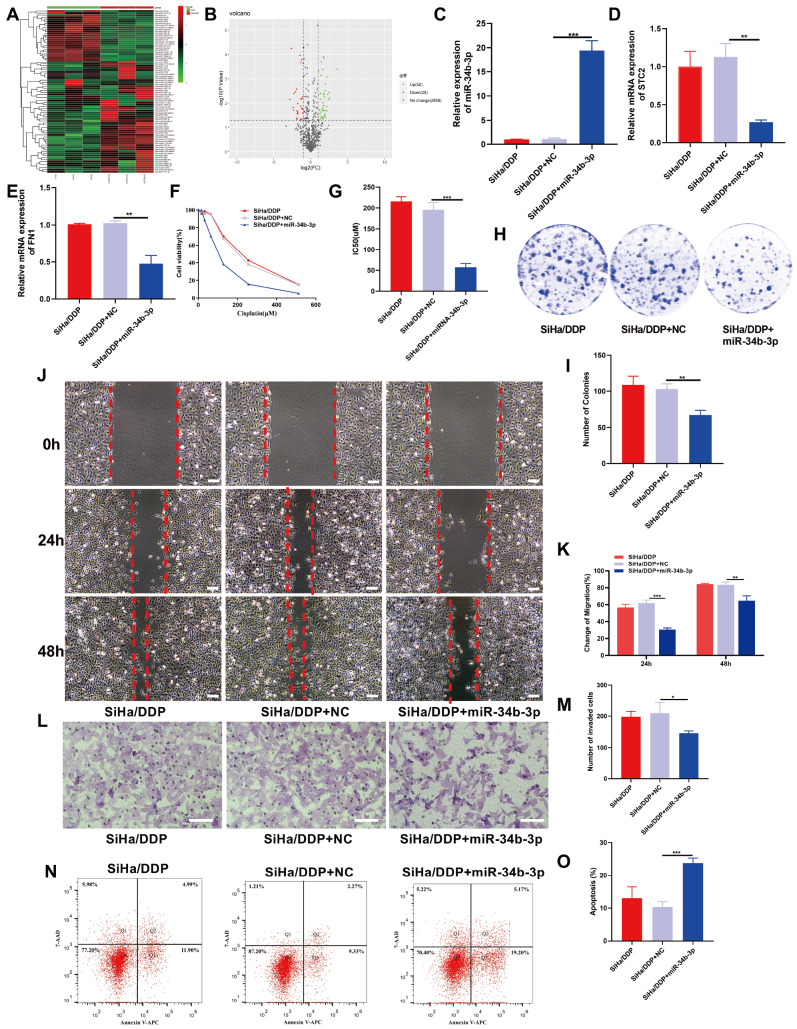
Figure 2 SiHa/DDP cells with low miR-34b-3 expression exhibit increased sensitivity to DDP, which inhibits cell proliferation and migration (A,B) miRNA expression patterns in SiHa and SiHa/DDP cells; volcano plot (A) and heatmap (B). (C–E) The expressions of miR-34b-3p (C), STC2 (D) and FN1 (E) in SiHa/DDP cells after transfection with miR-34b-3p mimics. (F,G) The effect of miR-34b-3p on the viability (F) and IC50 (G) of cisplatin in SiHa/DDP cells was assessed by CCK-8 assay. (H–M) Cell colony formation (H,I), migration (J,K) and invasion (L,M) were detected by colony formation, wound healing and transwell assays, respectively. (N,O) Flow cytometry was used to detect cell apoptosis. Scale bar: 50 μm. *P<0.05, **P<0.01, ***P<0.001 by Student’s t test.

Previous high-throughput sequencing analysis revealed that the expression of miR-34b-3p was significantly lower in the SiHa/DDP cells than in the SiHa cells, while the expression of STC2 was substantially greater ( Figure 2A,B) [9]. These findings were further validated by qRT-PCR and western blot analysis, which demonstrated a statistically significant difference in the expression levels of miR-34b-3p and STC2 between the SiHa/DDP and SiHa cells ( *P*<0.05).

To investigate the role of miR-34b-3p in CC cells, we transfected miR-34b-3p mimics into SiHa/DDP cells. qRT-PCR analysis confirmed that, compared with transfection with the miR-34b-3p NC, transfection with the miR-34b-3p mimics significantly upregulated the expression of miR-34b-3p. Furthermore, the expression of STC2 and FN1 was strongly downregulated upon miR-34b-3p overexpression ( *P*<0.05) ( Figure 2C‒E). Subsequently, we assessed the impact of upregulating miR-34b-3p on DDP-resistance in SiHa/DDP cells by CCK8 assay. The results indicated that overexpression of miR-34b-3p could notably reduce DDP-resistance in SiHa/DDP cells ( Figure 2F,G). In addition, the acquisition of drug resistance by tumor cells often coincides with enhanced proliferative, metastatic, and invasive abilities. Our findings revealed that overexpression of miR-34b-3p in SiHa/DDP cells compromised tumor formation, reduced metastatic ability, as indicated by the results of the wound healing assay, as well as the attenuated invasion ability determined by the Transwell assay ( Figure 2H‒M). Notably, overexpression of miR-34b-3p markedly increased the apoptosis rate of SiHa/DDP cells, as evidenced by the flow cytometry analysis ( Figure 2N,O).

### Role of *STC2* silencing in reducing DDP resistance and inhibiting malignant behavior of CC cells

**Figure 3:**
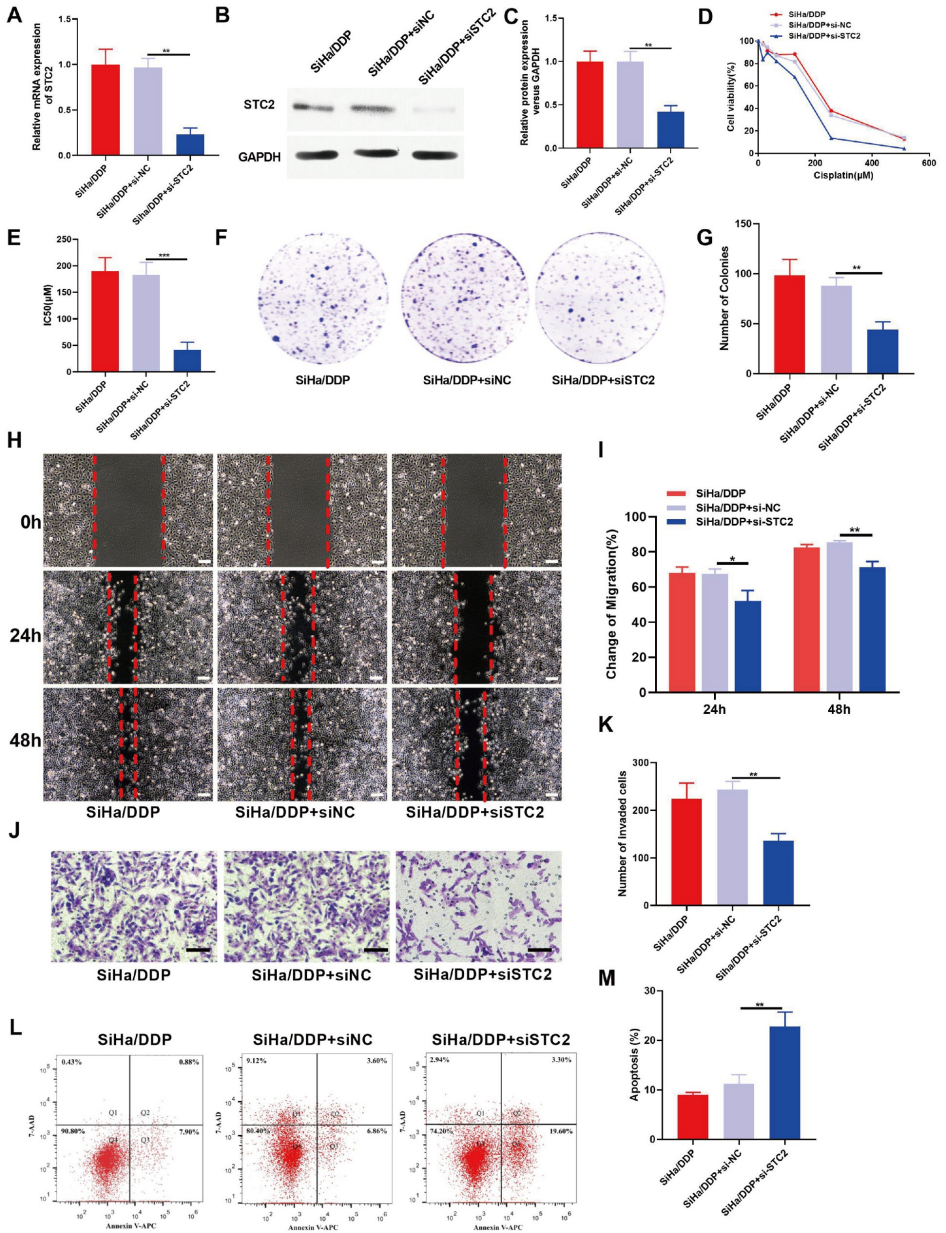
Figure 3 miR-34b-3p directly targets *STC2*, promoting chemoresistance, proliferation and migration in CC and reducing cell apoptosis (A–C) The transfection efficiency of siSTC2 was confirmed by qRT-PCR (A) and western blot analysis (B,C). (D,E) Cell viability (D) and the IC50 values (E) were detected by CCK-8 assay. (F–K) Cell colony formation (F,G), migration (H,I) and invasion (J,K) were detected by colony formation, wound healing and Transwell assays, respectively. (L,M) Flow cytometry was used to detect cell apoptosis. Scale bar: 50 μm. *P<0.05, **P<0.01, ***P<0.001 by Student’s t test.

Our previous investigations confirmed the high expression of STC2 in chemoresistant human CC cells and tissues, as well as its relationship with miR-34b-3p [9]. Here, we further explored the role of STC2 in CC cells by transfecting SiHa/DDP cells with small interfering RNA (siRNA) targeting *STC2*. The transfection efficiency was assessed by qRT-PCR and western blot analysis ( Figure 3A‒C). Subsequently, we assessed the impact of *STC2* silencing on DDP resistance in SiHa/DDP cells by CCK8 assay. The results demonstrated that silencing of *STC2* significantly inhibited cell resistance to DDP ( Figure 3D,E). Furthermore, functional assays, including colony formation, wound healing, and Transwell assays, were conducted to evaluate the effect of *STC2* silencing on SiHa/DDP cell behaviors. Notably, the results indicated that silencing of *STC2* effectively inhibited the colony formation, migration, and invasion abilities of SiHa/DDP cells ( Figure 3F‒K). Moreover, flow cytometry analysis revealed that silencing of *STC2* markedly enhanced cell apoptosis of SiHa/DDP cells ( Figure 3L,M).

### Role and downstream target analysis of FN1 in CC chemoresistance

**Figure 4:**
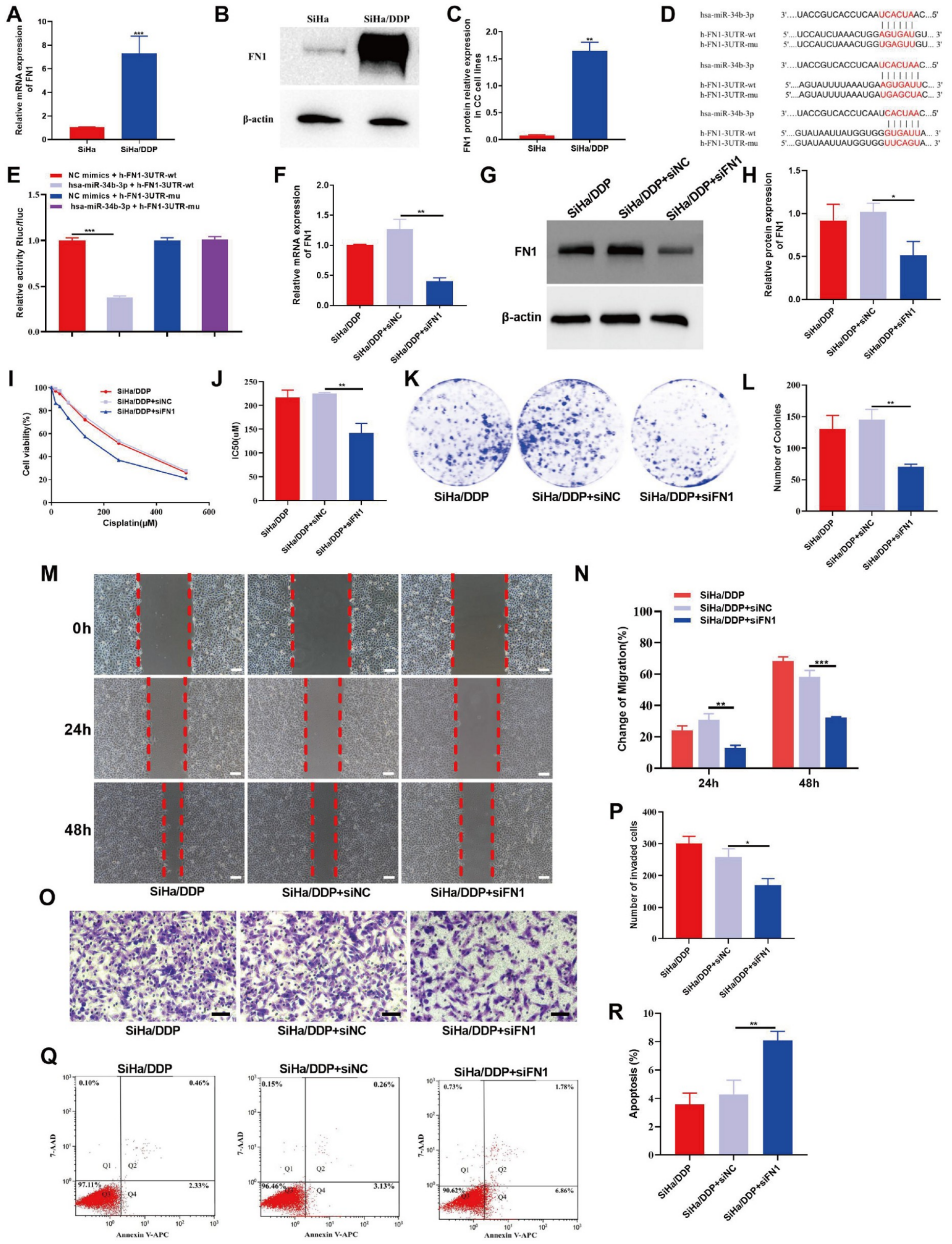
Figure 4 miR-34b-3p directly targets *FN1*, promoting chemoresistance, proliferation and migration in CC and reducing cell apoptosis (A–D) The expressions of FN1 in SiHa and SiHa/DDP cells were determined by qRT-PCR (A) and western blot analysis (B,C). (D) The binding sites between FN1-wild type/mutant type and miR-34b-3p. (E) The targeting relationship between miR-34b-3p and FN1 was verified by dual luciferase reporter assay. The transfection efficiency of siFN1 was confirmed by qRT-PCR (F) and western blot analysis (G,H). (I,J) Cell viability (I) and the IC50 (J) were detected by CCK-8 assay. (K–P) Cell colony formation (K,L), migration (M,N) and invasion (O,P) were detected by colony formation, wound healing and transwell assays, respectively. (Q,R) Flow cytometry was used to detect cell apoptosis. Scale bar: 50 μm. *P<0.05, **P<0.01, ***P<0.001 by Student’s t test.

In addition, overexpression of miR-34b-3p mimics appreciably reduced the expression of FN1 ( Figure 2E). Compared to SiHa cells, SiHa/DDP cells showed significantly greater expression of FN1 ( Figure 4A‒C). Further bioinformatics analysis indicated that FN1 was negatively related to miR-34b-3p, suggesting that *FN1* is an important downstream target gene of miR-34b-3p ( Figure 4D). Luciferase reporter assays also confirmed the interaction between FN1-WT or FN1-Mut and miR-34b-3p mimics or the NC ( Figure 4E). Cell function experiments were also conducted to explore the potential function of FN1. Knockdown of *FN1* in SiHa/DDP cells using specific siRNAs resulted in a significant reduction in cell resistance to DDP, as demonstrated by CCK-8 assay ( Figure 4F‒J). Colony formation assay indicated that knockdown of *FN1* decreased the colony formation ability of the cells ( Figure 4K,L). Wound healing and transwell assays revealed that silencing of *FN1* in SiHa/DDP cells significantly reduced cell migration and invasion abilities ( Figure 4M‒P). Additionally, flow cytometry analysis demonstrated that knockdown of *FN1* promoted apoptosis of SiHa/DDP cells ( Figure 4Q,R). Overall, these findings highlight the important role of FN1 in the development and chemoresistance of CC.

### STC2 and FN1 partially reverse the effects of miR-34b-3p against chemoresistance in CC cells

**Figure 5:**
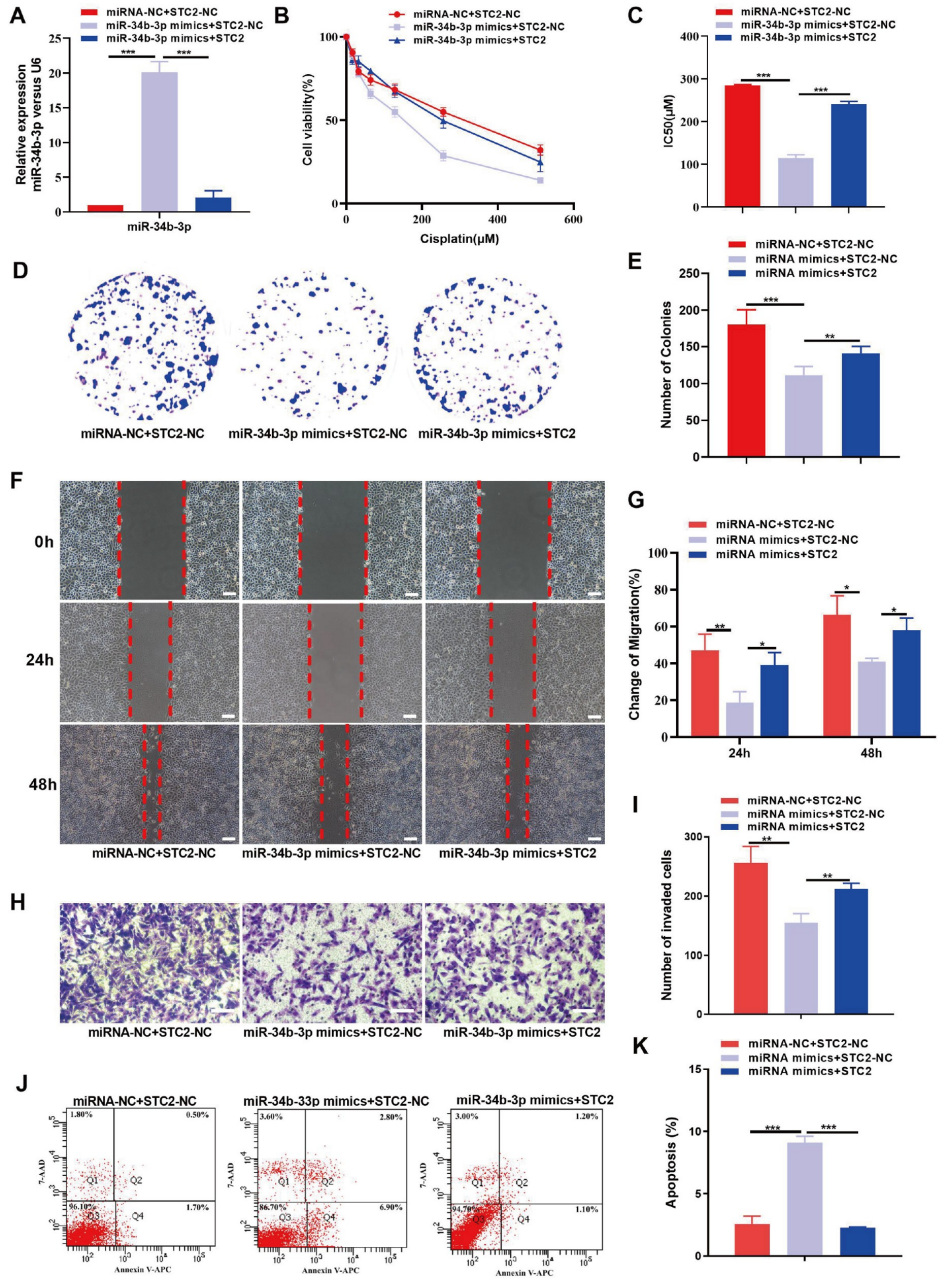
Figure 5 STC2 partially reverses the modulatory effect of miR-34b-3p on the DDP-resistant, proliferative, migratory and apoptotic potential of CC cells SiHa/DDP cells were transfected with miR-NC+pcDNA-NC, miR-34b-3p mimics+pcDNA-NC, or miR-34b-3p mimics+pcDNA-STC2. (A) Transfection efficiency was confirmed by qRT-PCR. (B,C) CCK8 assay was performed to determine the cell viability (B) and IC50 (C). (D–I) Colony formation, wound healing and Transwell assays were performed to evaluate colony formation (D,E), migration (F,G) and invasion (H,I), respectively. (J,K) Flow cytometry was performed to detect cell apoptosis. Scale bar: 50 μm. *P<0.05, **P<0.01, ***P<0.001 by Student’s t test.

**Figure 6:**
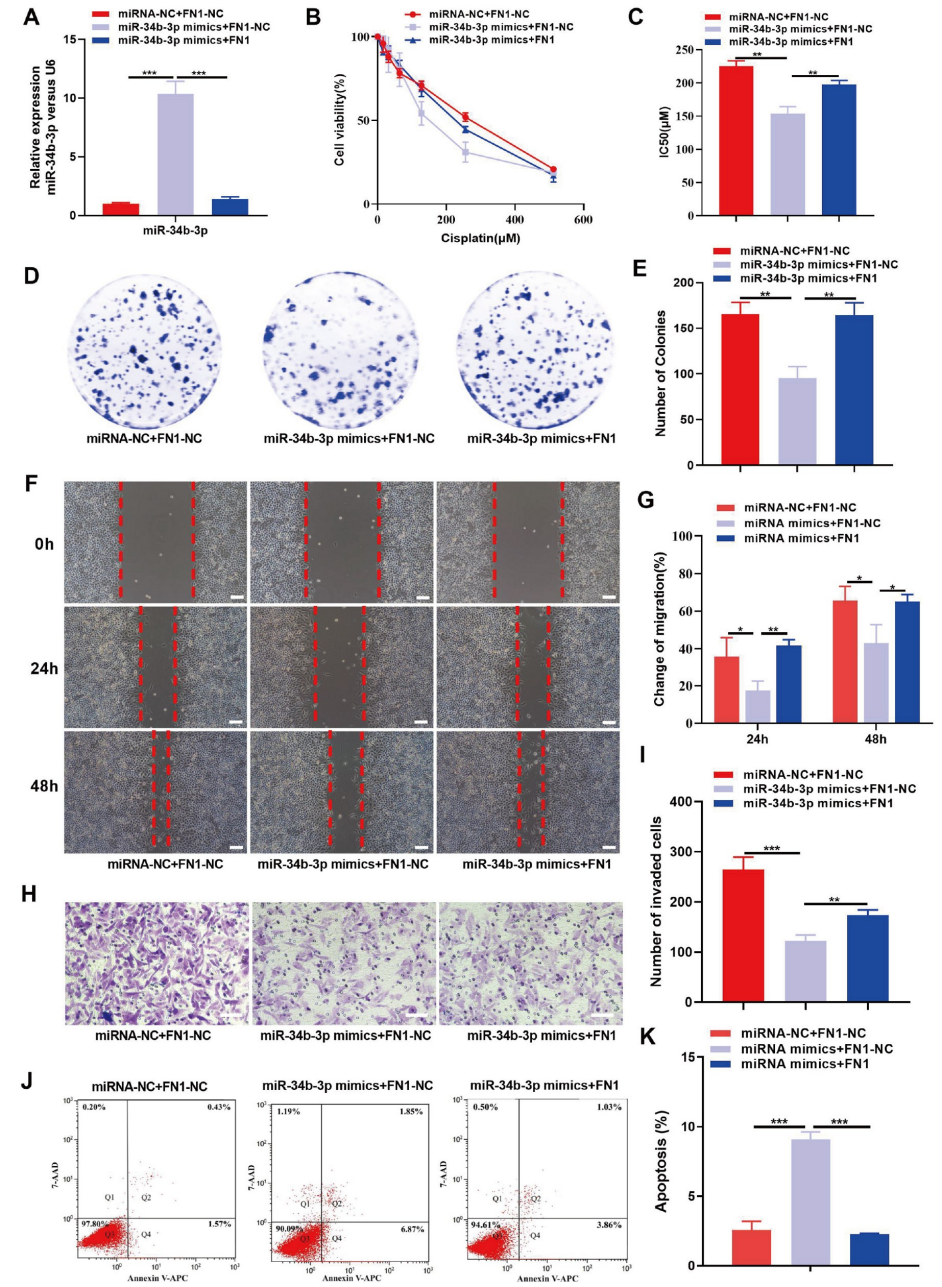
Figure 6 FN1 partially reverses the modulatory effect of miR-34b-3p on the DDP-resistant, proliferative, migratory and apoptotic potential of CC cells SiHa/DDP cells were transfected with miR-NC+pcDNA-NC, miR-34b-3p mimics+pcDNA-NC, or miR-34b-3p mimics+pcDNA-FN1. (A) Transfection efficiency was confirmed by qRT-PCR. (B,C) CCK8 assay was performed to determine the cell viability (B) and IC50 (C). (D–I) Colony formation, wound healing and Transwell assays were performed to evaluate colony formation (D,E), migration (F,G) and invasion (H,I), respectively. (J,K) Flow cytometry was performed to detect cell apoptosis. Scale bar: 50 μm. *P<0.05, **P<0.01, ***P<0.001 by Student’s t test.

To investigate whether STC2 or FN1 can reverse the effects of miR-34b-3p against chemoresistance and malignant behaviors in CC, we examined the biological behaviors of CC cells coexpressing miR-34b-3p mimics and STC2 (or miR-34b-3p mimics and FN1). The transfection efficiency was confirmed by qRT-PCR, which showed that the upregulation of STC2 (or FN1) significantly reduced the expression of miR-34b-3p ( Figures 5A and 6A). The results of CCK-8 assay demonstrated that overexpression of STC2 (or FN1) partially reversed the DDP-sensitivity induced by miR-34b-3p mimic in SiHa/DDP cells ( Figures 5B,C and 6B,C). Additionally, the colony formation, migration, and invasion abilities of SiHa/DDP cells, which were inhibited by miR-34b-3p mimics, were partially rescued by overexpression of STC2 (or FN1) ( Figures 5D‒I and 6D‒I). Flow cytometry analysis further showed that upregulation of miR-34b-3p stimulated the apoptosis of SiHa/DDP cells, and these effects were partially reversed by overexpression of STC2 (or FN1) ( Figures 5J,K and 6J,K). These findings suggest that miR-34b-3p may effect DDP-resistance by regulating STC2 or FN1 in SiHa/DDP cells.

## Discussion

In recent years, the advancement of high-throughput sequencing technology has led to the discovery of more miRNAs. These small RNA molecules have attracted increased attention due to their crucial roles in disease-related biological processes. Notably, miRNAs act as posttranscriptional regulators of gene expression, with each miRNA capable of targeting multiple genes and each gene being regulated by multiple miRNAs [10]. Numerous studies have demonstrated the involvement of miRNAs in various diseases, particularly in cancer [11]. Furthermore, miRNAs have been implicated in the development of resistance to cancer chemotherapy. For instance, Wang *et al*. [12] revealed that miR-149-3p promoted resistance to DDP in ovarian cancer by regulating CDKN1A and TIMP2. Likewise, Li *et al*. [13] identified a crucial role of miR-155-5p in paclitaxel resistance in breast cancer. Our previous sequencing analysis indicated significant downregulation of miR-34b-3p in both chemoresistant CC tissues and cells [9]. However, the underlying molecular mechanisms by which miR-34b-3p affects chemoresistance in CC patients have not been determined.

In this study, we initially focused on characterizing the drug resistance and biological functions of SiHa/DDP cells, a chemoresistant CC cell line model that we established. Our findings revealed that SiHa/DDP cells showed significantly increased resistance index to DDP, enhanced cell proliferation, migration and invasion, and reduced apoptosis compared to parental SiHa cells. Moreover, we observed upregulation of the expressions of drug resistance-related genes, including *MDR* [14], *GST* [15] and *ERCC* [16]. This model served as the foundation for subsequent whole-transcriptome sequencing of both cell types and our investigation into the mechanisms underlying DDP resistance in CC. miR-34b-3p, which is involved in various physiological processes, including chemoresistance, cell proliferation, cell death, cell migration, and cell invasion, was found to be dysregulated in cancers. Several studies have proven the downregulation of miR-34b-3p in small-cell lung cancer [17], breast cancer [18] and prostate cancer [19] and its involvement in chemoresistance in breast cancer [20]. Sergio *et al*. [21] examined the importance and function of miR-34b-3p in CC and reported that it is expressed at a low level and inhibits cell proliferation and migration. Nonetheless, the role of miR-34b-3p in chemoresistance in CC remains unclear. We validated the expression level of miR-34b-3p [9], which is consistent with the sequencing results. Functional experiments revealed that miR-34b-3p can suppress DDP-resistance, cell proliferation, migration and invasion in cervical carcinoma cells and promote apoptosis. The biological functions of miRNAs involve binding to the 3′-UTR of the target mRNAs, consequently regulating their transcription and translation [5]. To identify the downstream target genes of miR-34b-3p, we utilized sequencing data and conducted an online analysis, which revealed the interaction sites of miR-34b-3p with *STC2* and *FN1*. Subsequent mechanistic experiments confirmed the important role of miR-34b-3p in regulating FN1 and STC2. Collectively, our findings indicate that miR-34b-3p plays a role in cancer inhibition in the chemoresistance and progression of CC. Elucidating the role of miR-34b-3p in promoting chemoresistance may facilitate improvements in treatment outcomes, early diagnosis, prognosis, and the development of better CC treatment regimens.

The direct targeting of *FN1* and *STC2* by miR-34b-3p in CC has been confirmed. In addition, overexpression of FN1 or STC2 partially reversed the inhibitory effects of miR-34b-3p on cell growth and resistance to the chemical drug DDP *in vitro*. FN1, a member of the FN family, is extensively expressed in various cells and plays a crucial role in cell adhesion and migration [22]. It has been implicated in the development of gastric cancer [23], thyroid cancer [24], non-small cell lung cancer [25], and platinum resistance in ovarian cancer [26]. In our study, we observed high expression of FN1 in SiHa/DDP cells. Knockdown of *FN1* increased cell sensitivity to DDP, reduced cell migration, invasion and proliferation activities, and enhanced cell apoptosis. STC2 is a glycoprotein hormone involved in regulating calcium and phosphate homeostasis, as well as in responding to glutamine or glucose deprivation, hypoxia and endoplasmic reticulum stress [ 27– 29]. Recent studies have shown that elevated STC2 level is associated with tumor proliferation, invasion, migration, chemotherapy resistance, and poor prognosis [ 30– 33]. Our study further confirmed the oncogenic role of STC2 in promoting tumor cell proliferation, migration, invasion, and resistance to DDP in CC.

**Figure 7:**
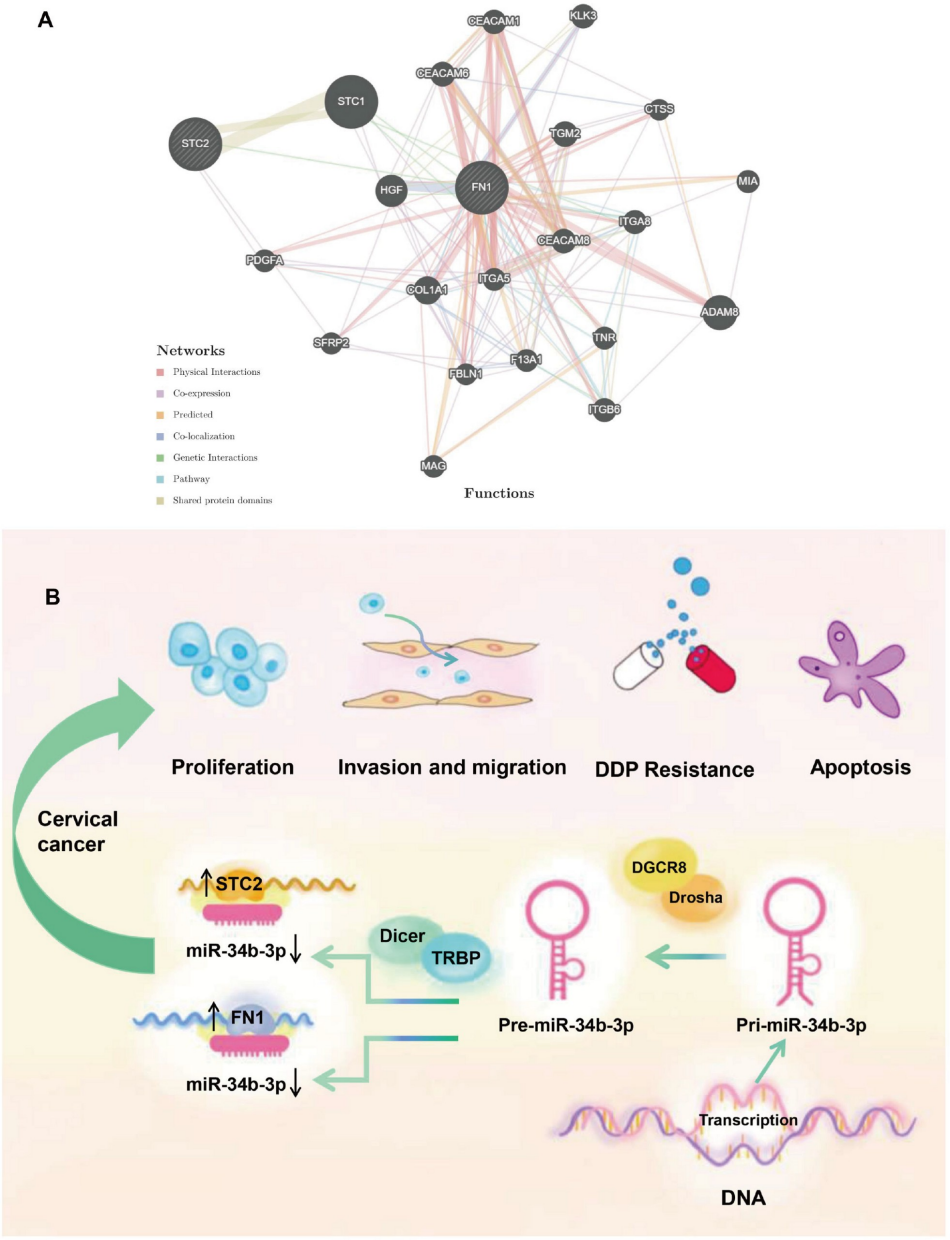
Figure 7 Summary of the regulatory mechanism of miR-34b-3p in CC (A) Network model: the lines signify the potential interactions between genes obtained from GeneMANIA (http://genemania.org/). (B) miR-34b-3p affects the chemoresistance and progression of CC by regulating STC2 and FN1.

Nevertheless, it should be acknowledged that this study has several limitations. First, *in vivo* confirmation of the effect of miR-34b-3p on CC chemoresistance was not performed, which is necessary in future investigations. Additionally, our data analysis suggested that miR-34b-3p, STC2, or FN1 may influence DDP-resistance in CC through specific signaling pathways. Previous studies have demonstrated that both FN1 and STC2 can activate signaling pathways such as the PI3K/Akt [ 34, 35] and MAPK [ 30, 36] pathways, which are known to promote tumorigenesis and development. For instance, Yuan *et al*. [37] reported that elevated level of STC2 activates the PI3K/AKT pathway, leading to increased expression of the drug-resistant protein P-gp and enhanced resistance of colorectal cancer cells to oxaliplatin. Similarly, Yoshihara *et al*. [26] showed that ovarian-associated mesothelial cells induce and activate the FN1/Akt signaling pathway, thereby promoting the resistance of ovarian cancer peritoneal metastases to DDP. Protein interaction analysis revealed potential crosstalk between STC2 and FN1, indicating possible coordination of downstream pathways ( Figure 7A). Consequently, it is plausible that miR-34b-3p modulates the initiation, development, and chemoresistance of CC by regulating downstream signaling pathways through the regulation of FN1 and STC2 expression ( Figure 7B). However, further investigations are warranted to elucidate the underlying molecular mechanisms of this intricate regulatory network.

In summary, our study provides experimental evidence supporting the role of miR-34b-3p in negatively regulating DDP-resistance in CC through the regulation of its target genes *STC2* and *FN1*. These findings suggest that therapeutic interventions targeting miR-34b-3p and its downstream genes *STC2* and *FN1* could be potential strategies for overcoming chemoresistance in CC.
